# Understanding stress in patients with multiple sclerosis: The joint predictive role of disease characteristics and emotion regulation processes

**DOI:** 10.1192/j.eurpsy.2021.1241

**Published:** 2021-08-13

**Authors:** T. Carvalho, A. Sousa-Mendes, C. Gomes, C. Guedes

**Affiliations:** 1 Psychology, Instituto Superior Miguel Torga, Coimbra, Portugal; 2 Faculty Of Psychology And Educational Sciences, University Of Coimbra, Center for Research in Neuropsychology and Cognitive-Behavioral Intervention (CINEICC), Coimbra, Portugal; 3 -, Clínica de Saúde Psiquiátrica de Coimbra – Casa da Oliveira, Coimbra, Portugal

**Keywords:** Multiple sclerosis, predictive model, clinical characteristics of multiple sclerosis, emotion regulation processes

## Abstract

**Introduction:**

Multiple sclerosis (MS) is a chronic inflammatory, demyelinating, and neurodegenerative disease of the central nervous system. This condition is enhanced by stress. In turn, stress symptoms are a risk factor for the onset and progression of MS. However, knowledge about predictors of stress in patients with MS is scarce.

**Objectives:**

This preliminary study aimed to verify whether the number of relapses, fatigue, physical disability (MS characteristics), experiential avoidance and self-judgment (emotion regulation processes) predict stress symptoms in patients diagnosed with MS.

**Methods:**

A convenience sample of 101 patients diagnosed with MS and without other neurological diseases participated in this study. Participants completed the Depression Scale of the Depression, Anxiety and Stress Scales-21, Analog Fatigue Scale, World Health Organization Disability Assessment Schedule-12, Acceptance and Action Questionnaire-II, and Self-Judgment Subscale of the Self-Compassion Scale.

**Results:**

All predictors initially hypothesized and years of education have significant correlations with stress symptoms. Simple linear regression analyses showed that the variables significantly predicted stress symptoms and were, therefore, included in the multiple linear regression model. This model explained 51.8% of the variance of the stress symptoms and showed that years of education, the number of relapses, fatigue, and experiential avoidance significantly predicted those symptoms.

**Conclusions:**

The promotion of mental health mental in patients with MS must develop functional skills to deal with stress induced by years of education (possibly responsible for the degree of awareness about MS and its consequences), recurrence of relapses and fatigue, and should minimize emotion regulation strategies focused on experiential avoidance.

